# Supplementary material for: Comparative multiomics analysis of cell physiological state after culture in a basket bioreactor
Source: Sci Rep. 2022 Nov 23;12:20161. doi: 10.1038/s41598-022-24687-4 (PMC9686226; doi:10.1038/s41598-022-24687-4)

88505841:+@NW_023666050.1:88509061:88509117:+@NW_023666050.1:88512190:88512275:+@NW_023666[illegible]

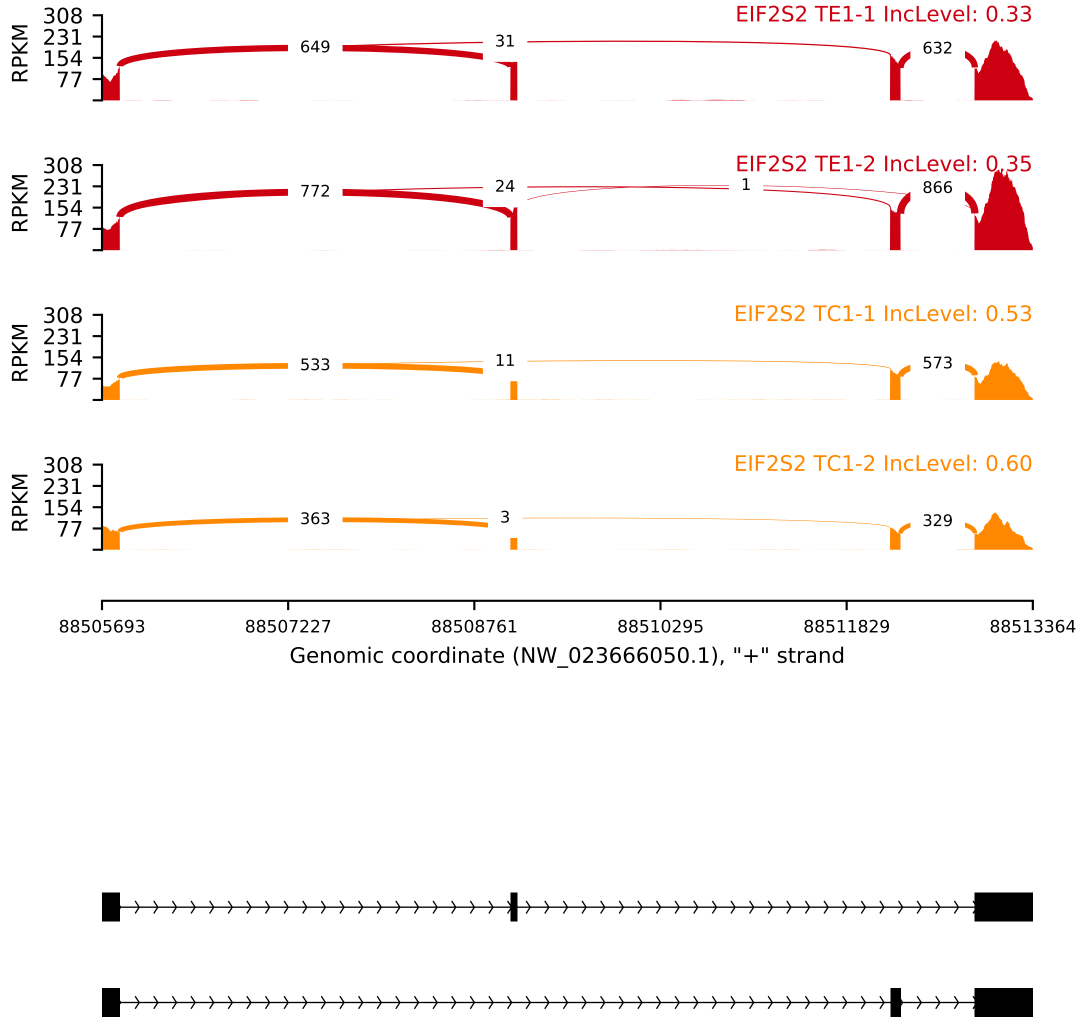

Supplement: Supplementary file 1 — Supplementary Information 1. [file 41598_2022_24687_MOESM1_ESM.zip › raw data/Transcriptomic raw data/7.AS/2.dasplot/TE1vsTC1/MXE/16_EIF2S2_NW_023666050.1_88505693_88505841_+@NW_023666050.1_88509061_88509117_+@NW_023666050.1_88512190_88512275_+@NW_023666050.1_88512886_88513365_+.pdf]
